# Methylation in the CHH Context Allows to Predict Recombination in Rice

**DOI:** 10.3390/ijms232012505

**Published:** 2022-10-19

**Authors:** Mauricio Peñuela, Jenny Johana Gallo-Franco, Jorge Finke, Camilo Rocha, Anestis Gkanogiannis, Thaura Ghneim-Herrera, Mathias Lorieux

**Affiliations:** 1 iÓMICAS, Facultad de Ingeniería y Ciencias, Pontificia Universidad Javeriana, Cali 760031, Colombia; 2 The Alliance of Bioversity International and the International Center for Tropical Agriculture (CIAT), Palmira 763537, Colombia; 3 Departamento de Ciencias Biológicas, Universidad ICESI, Cali 760031, Colombia; 4 DIADE, University of Montpellier, CIRAD, IRD, 34394 Montpellier, France

**Keywords:** epigenetic, DNA methylation, bisulfite sequencing, machine learning, modeling

## Abstract

DNA methylation is the most studied epigenetic trait. It is considered a key factor in regulating plant development and physiology, and has been associated with the regulation of several genomic features, including transposon silencing, regulation of gene expression, and recombination rates. Nonetheless, understanding the relation between DNA methylation and recombination rates remains a challenge. This work explores the association between recombination rates and DNA methylation for two commercial rice varieties. The results show negative correlations between recombination rates and methylated cytosine counts for all contexts tested at the same time, and for CG and CHG contexts independently. In contrast, a positive correlation between recombination rates and methylated cytosine count is reported in CHH contexts. Similar behavior is observed when considering only methylated cytosines within genes, transposons, and retrotransposons. Moreover, it is shown that the centromere region strongly affects the relationship between recombination rates and methylation. Finally, machine learning regression models are applied to predict recombination using the count of methylated cytosines in the CHH context as the entrance feature. These findings shed light on the understanding of the recombination landscape of rice and represent a reference framework for future studies in rice breeding, genetics, and epigenetics.

## 1. Introduction

Meiotic recombination is recognized as a key process in genetics. During this process, maternally and paternally inherited homologous chromosomes exchange information by gene conversion or crossing over to create novel allelic combinations. Recombination is widely recognized for its role in promoting diversity to respond to continually shifting environments, in addition to preventing the build-up of genetic load by decoupling linked deleterious and beneficial variants [1]. However, meiotic recombination between homologous chromosomes is restricted by the number and location of crossover sites per chromosome. The crossover distribution and frequency along the genome are uneven, especially in plants [2]. Sites with high recombination rates have been linked to subtelomeric regions that are generally hypomethylated and have high gene and DNA transposon frequencies. In contrast, recombination is suppressed in the centromeric region, which is characterized by high frequencies of long terminal repeat retroelements and few genes [3].

The role of chromatin structure and DNA methylation in determining recombination rates has been previously reported. For example, high levels of histone H3 acetylation in *Arabidopsis* mutants were associated with changes in the crossover frequencies [4]. Likewise, studies using *met1* and *ddm1* mutants, which are globally hypomethylated, showed regional remodeling of crossover frequencies with increased recombination in chromosome arms and decreased recombination in the pericentromeric region [5,6]. However, understanding how the DNA methylation patterns affect the recombination rates remains an open challenge.

In plants, DNA methylation occurs at cytosine nucleotides in all the sequence contexts CG, CHG, and CHH (H = C, T, or A). DNA methylation is a stable mark inherited from generation to generation and a crucial factor for plant development [7]. DNA methylation, in combination with histone and non-histone protein modifications, defines chromatin structure and accessibility, which helps to regulate gene expression, transposon silencing, chromosome interactions, and trait inheritance [8]. Several studies have shown that sexual reproduction in plants involves the reprogramming of DNA methylation patterns [9].

The methylation dynamics for each sequence context is determined by different mechanisms and related to specific biological functions [8]. The maintenance mechanism of plant DNA methylation depends on the context and is mediated by different enzymes. For example, in *Arabidopsis thaliana*, CG cytosine methylation is maintained by *MET1*, in a semiconservative manner in the DNA replication process, while CHG methylation is maintained by *CMT3* and *CMT2*, which enables the propagation of methylation through a positive feedback loop together with the *H3K9me2* in the cell division process. Meanwhile, CHH methylation is maintained by *DRM2* or *CMT2*, depending on the genomic region [8]. De novo methylation is carried out by *CMT2* for CHG and CHH context [9], and the *RdDM* pathway for all sequence contexts [9]. This process is not the same for all plants. In rice, CG cytosine methylation is carried out by two related genes *OsMET1-1* and *OsMET1-2,* with a possible redundant function, while *OsCMT3a* is the only functional ortholog of *CMT3* involved in CHG methylation during replication. For CHH methylation, no associated gene has yet been reported. There is some evidence that suggests that *OsCMT2* is closely related to *CMT2* and may play a role in CHH methylation [10]. More research on methylation and demethylation events and their precursors will be necessary to clarify these mechanisms.

Identifying factors influencing the meiotic recombination rates is important for breeders interested in transferring genes from one variety to another through crosses. Thus, developing new allelic combinations that allow breeders to meet the needs present in agricultural systems. Recently, a number of studies have addressed this issue and have developed different types of strategies to discover where crossovers occur most frequently and try to predict them. For example, Liu et al. [11] developed a predictor of recombination hot/cold spots in yeast using a machine learning approach combined with principal component analysis. Moreover, Demirci et al. [12] explored DNA sequence and shape features to train machine learning models for predicting crossover occurrence in *Arabidopsis*, maize, tomato, and rice. Moreover, Adrion et al. [13] used recurrent neural networks, a deep learning method for estimating genome-wide recombination in a natural population of African *Drosophila melanogaster.*

In recent years, rice has been a model monocotyledonous plant for understanding the methylation process because it is highly homozygous and self-pollinating. In addition, rice is of great importance in food security since half of the world’s population depends on it as daily food [14]. However, few studies have analyzed methylation patterns in relation to recombination rates in rice. For instance, Habu et al. [15] developed an experiment crossing methylated and unmethylated rice varieties and concluded that the position and frequency of meiotic recombination in rice centromeric heterochromatin are regulated by the epigenetic state of the chromatin. Likewise, Choi et al. [16] explore how transposable elements interact with host plant epigenetics. They suggest that high levels of methylation at these elements have a role in suppressing deleterious ectopic recombination. Nevertheless, none of these studies have explored in detail how the methylation contexts are related with recombination rates.

In this work, the relationship between chromosomal recombination rates and different methylation contexts is explored by using *Oryza sativa* as a model. The focus is on the following objectives: (1) To estimate the correlation between recombination and methylation in all contexts, (2) to describe the effect of methylation within genes, transposons, and retrotransposons with respect to recombination, and (3) to implement a machine learning model to predict recombination based on methylation data. The results provide evidence that recombination can be described by methylation in the context of CHH, regardless of whether it is outside or inside genes, transposons, and retrotransposons. Consequently, the use of machine learning models to predict chromosomal recombination rates in rice cultivars using CHH methylation is proposed.

## 2. Results and Discussion

**Figure 1:**
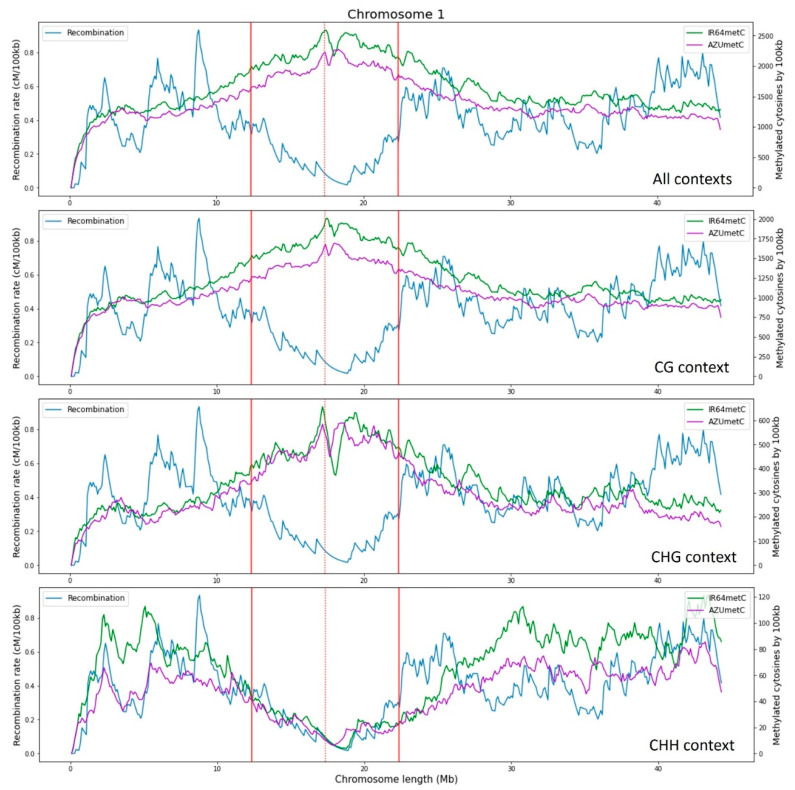
Recombination and methylated cytosines through Chromosome 1 for the rice varieties IR64 and Azucena. The centromere is represented by a red dotted line and the influence of the centromere region by solid red lines.

**Figure 2:**
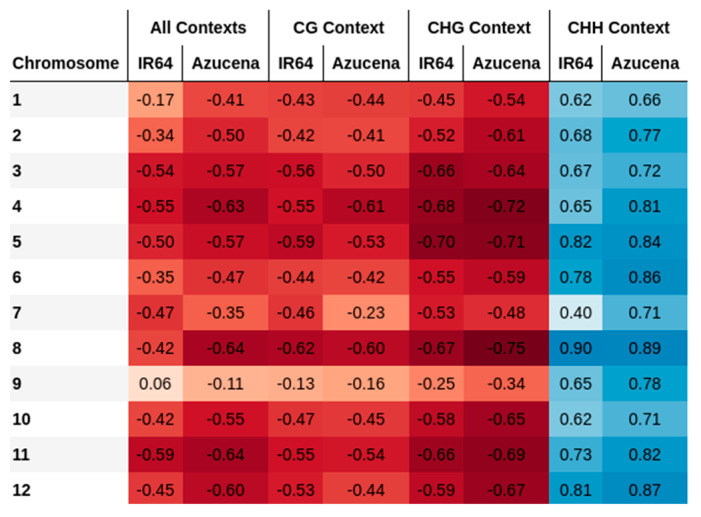
Correlations between recombination rates and the count of methylated cytosines for rice varieties IR64 and Azucena. Blue and red colors correspond to positive and negative correlations, respectively. The higher the correlation value, the higher the color intensity.

In this study, the correlation between recombination rates and the methylated cytosine counts for all chromosomes in two rice cultivars is evaluated (Figure 1 and Figure 2). The correlation values are, on average, −0.44 ± 0.17 for all chromosomes of both varieties, with higher values in the centromere region. Similar results in rice were previously described by Yan et al. [17], revealing that DNA methylation patterns in the centromere are shaped by the DNA sequence and the centromeric domains. Habu et al. [15] described how artificial chromatin modification can vary the frequency of meiotic recombination. Overall, high levels of methylation in heterochromatin regions near the centromeres have been reported as a common pattern, where meiotic recombination is repressed. In the same way, recombination-free regions around centromeres are likely to be important for normal centromere function during meiosis [15,18].

**Figure 3:**
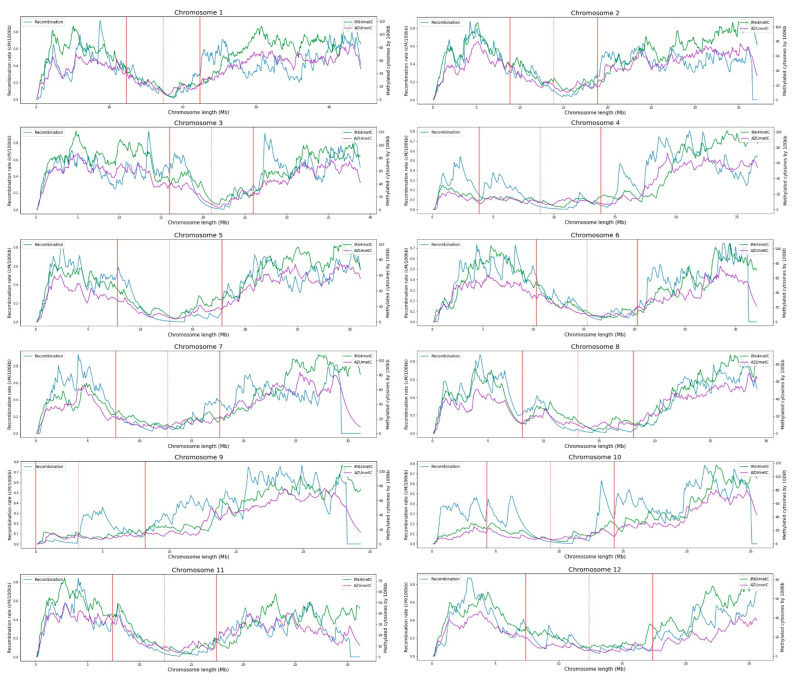
Distribution of methylated cytosines in CHH context in the twelve rice chromosomes for the IR64 and Azucena varieties, in comparison with the chromosomal recombination between these two varieties. The centromere is represented by a red dotted line and the influence of the centromere region in recombination by solid red lines.

By evaluating the CG and CHG methylation contexts independently, a decrease in recombination rates with increasing methylated cytosines is reported. On the contrary, methylated cytosines in the CHH context increase with recombination rates showing a positive correlation (Figure 3). The opposite relationship between the methylation contexts of CG and CHH has been reported in rice by Li et al. [19], who identified the tendency towards hypermethylation in CG context, but hypomethylation in CHH.

The positive correlation between methylated cytosine count and recombination rates observed in the context of CHH is not clear when all methylation contexts are assessed together because the total number of methylated cytosines in the CG and CHG contexts was higher. This trend is observed for both varieties, IR64 and Azucena, where the methylation data and the alignment process have been obtained independently. The positive relationship between the CHH methylated cytosine count and recombination rates has been reported by Rodgers-Melnick et al. [1], who include the CHH methylation as a feature of a linear model to predict recombination in maize. It is unclear what role methylated cytosines play in the CHH context with respect to recombination.

Variability in DNA methylation can be heritable or reversible, and this can allow for phenotypic variation and rapid response to environmental changes. Even the degree of intraspecies epigenomic diversity can be correlated with climate and geographic origin [10]. It has been reported that CHH methylation could be related to fruit size in apples [20] and silencing transposons in sugar beets [21]. A potential role in *A. thaliana* seed dormancy, with increases in CHH methylation in seeds during seed development and a decrease during germination, has also been reported in [8]. These observations suggest the multiple roles that CHH methylation can play in plant genomes. Recently, Wang et al. [22] reported that CHH methylation levels are higher in rice reproductive organs, such as panicles and pistils, than in seedlings, suggesting a positive feedback loop between DNA methylation and RNA-directed DNA methylation activity involved in sexual reproduction.

**Figure 4:**
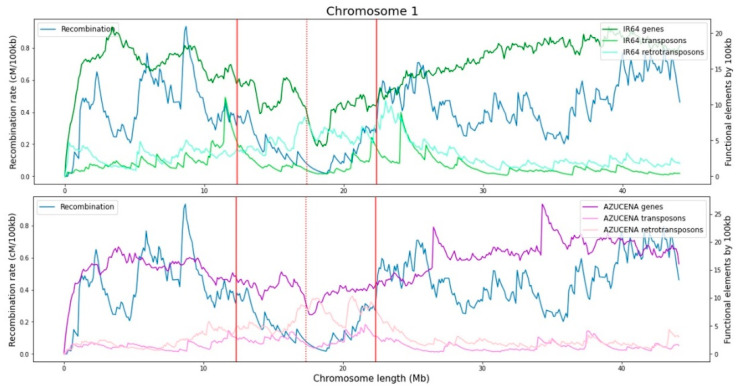
Genes, transposons, and retrotransposons compared to cross over recombination through chromosome 1 for rice varieties IR64 and Azucena. The centromere is represented by a red dotted line and the influence of the centromere region in recombination by solid red lines.

**Figure 5:**
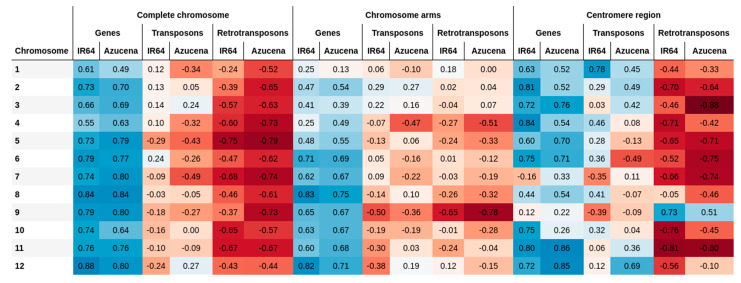
Correlations between recombination rates and the number of genes, transposons, and retrotransposons rates for rice varieties IR64 and Azucena. Blue and red colors correspond to positive and negative correlations, respectively. The higher the correlation value, the higher the color intensity.

The functional analysis performed with annotation data of genes, transposons, and retrotransposons for each variety, shows that the increment in the number of genes per window is correlated with recombination rates in the chromosomes of both varieties (Figure 4 and Figure 5). This positive trend has been previously evidenced in *Drosophila*, *A. thaliana*, yeast, finches, monkeyflowers, and dogs, with recombination hotspots typically located near the promoter regions of genes [23] and observed in the euchromatic regions of maize [24]. In contrast, a negative correlation between the number of transposons and retrotransposons has been found with respect to recombination rates across all chromosomes for both rice varieties. This can be explained by the abundance of such elements near the centromere where recombination rates are low. Similar results have been found by Tian et al. [25], who suggested that the rice genome is organized along recombinational gradients due to the negative correlation of recombination with transposable elements and positive one with gene densities.

Recombination tends to occur within and near genes and away from transposable elements. This may reflect the passive effects of recombination initiating in open chromatin [23]. Recent analyses of the localization of recombination at the fine scale tend to show negative correlations with local densities of repetitive elements. Actually, strong recombination suppression and a large accumulation of transposable elements are usual in pericentromeric regions [23]. For rice, this pattern is shared between *japonica* and *indica* groups [25]. There remains uncertainty about the directionality of cause and effect, the extent to which the correlation is driven by associations of both recombination and transposable elements with other factors, or why patterns differ among species and types of repetitive elements [23].

The count of methylated cytosines is assessed within genes, transposons, and retrotransposons and compared to recombination rates (Supplementary Materials Figures S1 and S2). The analysis shows that methylated cytosine count in genes, transposons, and retrotransposons is negatively correlated with recombination rates when evaluated for all contexts together (Supplementary Materials Figure S3). This indicates that methylation inside these entities is higher when recombination is lower. The same negative trend is observed when methylated cytosines are analyzed in CG and CHG contexts. Methylation events in transposons and retrotransposons are associated with the prevention of their expression and movement in chromosomes, which can be damageable to the organism and even deleterious [23,26]. It should be noted that these methylation events can also affect surrounding genomic regions [26], potentially influencing the methylation status of nearby genes. In genes, methylation usually occurs at the promoters or within the body of the transcribed gene, inhibiting their expression [8]. However, the methylated cytosines in the CHH context are also positively correlated with the recombination rates. This is a consequence of low CHH methylation near the centromere region and greater presence in the chromosome arms. Gallo-Franco et al. [27] reported high CHH methylation levels of transposable elements close to genes in rice, which supports the conclusion of Martin et al. [28] for grass species that long genes and genes close to transposable elements tend to have CHH islands more frequently. It could be hypothesized that the presence of these CHH islands is promoting the positive correlation between methylation and recombination in gene-rich regions.

**Figure 6:**
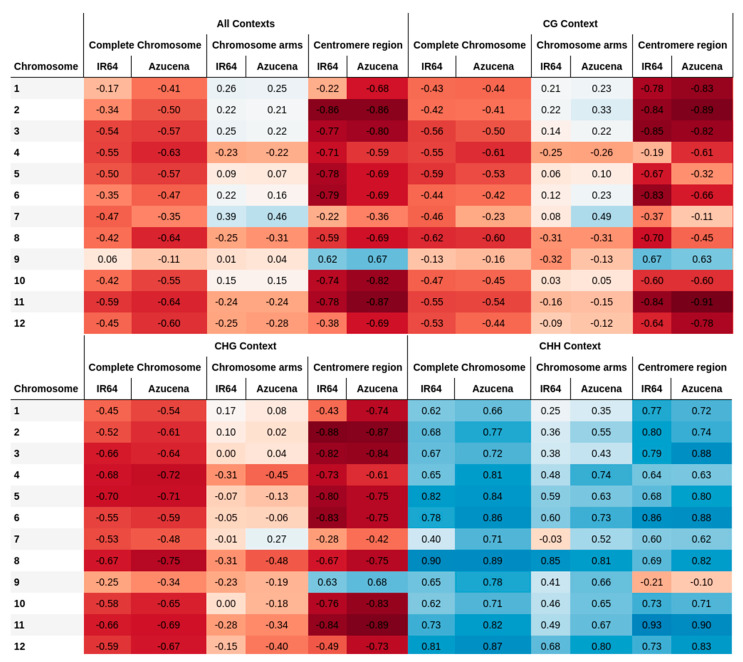
Correlations between recombination rates and the count of methylated cytosines for complete chromosomes, chromosome arms, and centromere region of rice varieties IR64 and Azucena. Blue and red colors correspond to positive and negative correlations, respectively. The higher the correlation value, the higher the color intensity.

Chromosomal regions close to the centromere have a high incidence on methylation. When only the chromosome arms are evaluated, correlation trends change, from being high negative to being negative, for all contexts evaluated together and for the CG and CHG contexts evaluated independently (Figure 6). For CHH methylation, the markedly positive correlation also decreases but is still positive. In the context centromere regions are evaluated, negative correlations are evidenced in all contexts when they are evaluated together and for CG and CHG contexts independently. These results are in agreement with the reported importance of DNA methylation for plant chromosomal interactions in pericentromeric regions [9]. They also agree with the results obtained by Habu et al. [15], who indicate that the position and frequency of meiotic recombination in the centromeric heterochromatin of rice are regulated by the epigenetic state of the chromatin. With respect to methylation in CHH contexts, the correlation of the centromere region is positive but weaker than that of the whole chromosome (Figure 6).

**Figure 7:**
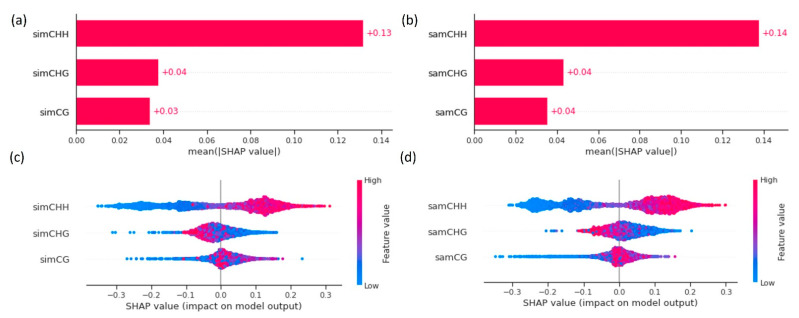
Shap values and contributions of features CG, CHG, and CHH to the prediction of recombination rates, using IR64 and Azucena data: (**a**) Shap summary plot of features for the IR64 variety; (**b**) Shap summary plot of features for the Azucena variety; (**c**) Shap values for the IR64 variety; and (**d**) Shap values for the Azucena variety.

The contributions of methylation in CG, CHG, and CHH contexts to predict recombination as features of machine learning models are assessed using the Shapley package. The results show a great contribution of CHH for the prediction of recombination and a low contribution of CG and CHG for both varieties (Figure 7). This agrees with the fact that the CHH context has the highest correlation values with respect to chromosome recombination rates, while the CG and CHG contexts have lower correlations. The Shap summary plot also shows the same trend, evidencing the strongest effect on recombination when the CHH values are higher.

Subsequently, the methylated cytosine count in the CHH context is used as a unique feature to evaluate regression algorithms of machine learning, because the performance of the model decreases when the other features are considered. The evaluation is carried out independently for each variety using the Lazy Predict package. The results show that the Extra Trees algorithm performed the best prediction (*R*^2^ = 0.57, *RMSE* = 0.01 for IR64; *R*^2^ = 0.69, *RMSE* = 0.01 for Azucena). Thus, this algorithm is used to develop the training and subsequent predictions.

**Figure 8:**
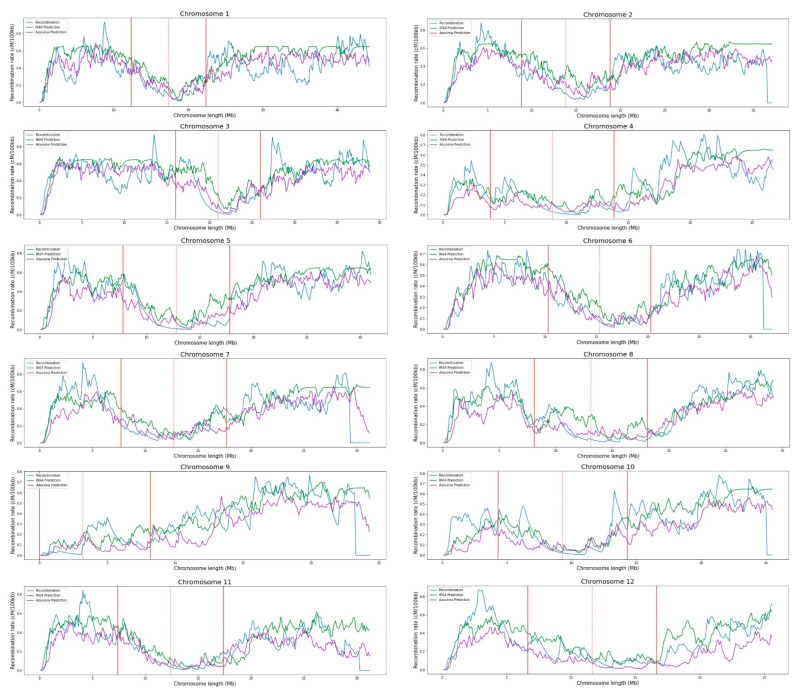
Recombination predictions between IR64 and Azucena varieties by the Extra Trees machine learning model using the count of methylated cytosines in the CHH context as a feature. Predictions on the IR64 manifold are made using Azucena methylation as the training dataset, and predictions on the Azucena manifold are made using IR64 methylation as the training dataset. The centromere is represented by a red dotted line and the influence of the centromere region in recombination by solid red lines.

Predictions on Azucena’s chromosomes, by training the Extra Trees algorithm with information from IR64, give an *R*^2^ of 0.32 ± 0.13 and an *MSE* of 0.02 ± 0.00, on average. Meanwhile, predictions on IR64’s chromosomes by training the Extra Trees algorithm with information from Azucena give an *R*^2^ of 0.21 ± 0.21 and an *MSE* of 0.03 ± 0.00, on average. In both cases, the average correlation values between predictions and recombination rates are 0.67 ± 0.06 for Azucena and 0.65 ± 0.07 for IR64, evidencing a positive trend (Table 1, Figure 8).

Several studies have focused on predicting recombination using machine learning. For example, Liu et al. [11] combined support vector machines with consensus feature dinucleotide-based autocross covariance to predict the recombination of hot/cold spots in yeast. Demirci et al. [12] used features, such as gene annotation, propeller, and helical twist, AT/TA dinucleotides, and CA dinucleotides to train machine learning models for predicting crossover occurrences in *Arabidopsis,* maize, rice, and tomato. More recently, Adrion et al. [13] proposed an approach to predict the recombination landscape in African populations of *Drosophila melanogaster* using deep learning with recurrent neural networks. For all cases, the results have been satisfactory according to the specific objective of each study, which demonstrates the power of machine learning approaches to predict complex traits such as chromosomal recombination.

The Extra Trees regression model makes it possible to predict chromosomal recombination using a single feature: The CHH methylated cytosine count. It is possible due to the high correlation between this feature and the recombination rates, which behaved similarly in all chromosomes. The model was trained on a dataset of one variety and was tested it on the other, performing two independent tests and finding that results were consistent (Figure 8). This opens a door for future studies. The evidence suggests that these models can be used to predict chromosomal recombination rates in any variety of *Oryza sativa* rice. This is because the two varieties used in this study, IR64 and Azucena, are highly distant genetically, belonging to the *indica* and *japonica* groups, respectively.

## 3. Materials and Methods

### 3.1. Recombination Rates

The recombination rates are estimated from an inter-subspecific segregating population of 212 F11 recombinant inbred lines (RIL). They are obtained by single seed descent, derived from the cross between the rice varieties IR64 (*indica* group) and Azucena (tropical *japonica* group), and genotyped using shallow Illumina sequencing (~2×) followed by imputation with NOISYmputer. Local recombination rates in cM/bp are calculated in sliding windows of 100 kb using MapDisto.

### 3.2. Plant Material and Growth Conditions for Methylation Experiment

Seeds of rice varieties IR64 and Azucena were germinated and grown in a growth chamber at 30 °C and 12:12 dark/light conditions for 10 days. Seedlings were transferred to a hydroponic medium with a Kimura B solution (pH 7) and Arnon micronutrients. Roots from three weeks-old seedlings were collected and stored at −80 °C. Total genomic DNA was extracted from frozen root tissue by CTAB 2X protocol with modifications [29]. Genomic DNA quality was evaluated on agarose gels, and DNA quantity was measured using a Nanodrop spectrophotometer (Thermo Fisher Scientific, Waltham, MA, USA).

### 3.3. Whole-Genome Bisulfite Sequencing and Data Analysis

Bisulfite-seq (BS-seq) libraries were made from genomic DNA isolated from IR64 and Azucena seedling roots. DNA from three independent seedlings for each genotype was pooled as one sample and sequenced. Bisulfite conversion of DNA, library construction, and sequencing were performed by CD Genomics (CD Genomics Inc., Shirley, New York, NY, USA). Raw data are available in the GenBank repository for IR64 (Accession number: SRR20325840) and Azucena (Accession number: SRR20325842). Basic statistics on the quality of the raw reads was done with the FastQC tool (https://www.bioinformatics.babraham.ac.uk/projects/fastqc/ (accessed on 5 September 2021)). Sequencing adapters and low-quality data of the sequencing data were removed by Trimmomatic (http://www.usadellab.org/cms/?page=trimmomatic (accessed 21 November 2021)). Cleaned data were aligned to the reference genomes reported in the GenBank repository for IR64 (Accession number: RWKJ00000000) and Azucena (Accession number: PKQC000000000) using Bismark v.0.16.3 [30] with default parameters. Only uniquely aligned reads were maintained. Methylation calling data obtained from Bismark were used for further analysis.

### 3.4. Comparison between Recombination Rates and Methylation Patterns

To compare the methylation patterns with the local recombination rates, the genomes were divided into 100 kb windows, in which the number of cytosines with a methylation level greater than 75% was calculated for each of the CG, CHG, and CHH contexts. Exponential smoothing with α = 0.1 was applied to the recombination and methylation data to remove noise associated with the abrupt change in the count of methylated cytosines in adjacent windows. Subsequently, a Pearson correlation analysis per chromosome was developed to evaluate the linear relationships between the recombination rates and the methylation patterns of both varieties.

### 3.5. Functional Evaluation

Gene, transposon, and retrotransposon annotation information from both varieties was used (Supplementary Materials Tables S1 and S2). Pearson correlation analyses were carried out between the number of genes, transposons, and retrotransposons with respect to recombination of the chromosome to investigate their relationship with the recombination landscape. Later, the start and end coordinates of these elements were used to extract the count of methylated cytosines inside them. New correlation analyses were performed to learn the trends between methylated cytosines for each context within these functional elements with respect to recombination. A differentiation between the centromere and non-centromere regions was also included.

### 3.6. Machine Learning Modeling

To assess the usefulness of methylation in predicting chromosome recombination, different machine learning approaches were explored. The total counts of methylated cytosines in windows of 100 kb belonging to the CG, CHG, and CHH contexts for each variety were evaluated as features for machine learning modeling using the Shapley package (https://shap.readthedocs.io/en/latest/index.html (accessed on 2 February 2022)). Subsequently, the performance of different machine learning models was evaluated using the LazyPredict package (https://pypi.org/project/lazypredict/ (accessed on 2 February 2022)). Exponential smoothing with α = 0.1 was applied to the data input before training the model and another one to the model output with α = 0.3. The coefficient of determination *R*^2^ and the root of the mean square error *RMSE* were used to evaluate the performance of the models. *MSE* was used for predictions. Pearson correlation analyses were also performed to discover general linear trends between the predictions and the experimental data. The resulting best model was fitted, and the information from the twelve chromosomes of one variety was used as a training dataset to predict the recombination rates in each of the twelve chromosomes of the other variety. All these analyses and the previous ones were run in Python.

## 4. Conclusions

This study reported on how methylated cytosines in the CHH context positively correlate with recombination rates in the twelve rice chromosomes of two genetically distant rice varieties: IR64 and Azucena. However, a negative correlation was found between methylation and recombination rates when only CG and CHG contexts were tested, as well as in the three methylation contexts together. For this case, the positive correlation of CHH was hidden due to the high number of methylated cytosines from the CG and CHG contexts. In addition, functional analysis showed that genes were positively correlated with recombination rates, unlike transposons and retrotransposons, which showed a negative correlation. The correlation between methylation and recombination suggests the same trends for the entire genome with respect to only methylation in genes, transposons, and retrotransposons. The influence of the centromere on methylation patterns and its correlation with recombination rates was evident, supporting the hypothesis that the position and frequency of meiotic recombination in rice centromeric heterochromatin are regulated by the epigenetic state of the chromatin. Finally, a machine learning model was proposed and trained using the CHH methylated cytosine count to predict recombination rates, which obtained consistent results in two independent data sets. This suggests that the extraction of methylation data and the use of machine learning models in future studies is a promising path to focus on predicting recombination rates using the count of CHH-methylated cytosines in rice as a feature. Colleagues are invited to explore how the counting of CHH-methylated cytosines in other species behaves with respect to chromosomal recombination.

## Figures and Tables

**Table 1:** Performance of chromosome recombination rates predictions of IR64 and Azucena rice varieties using the Extra Trees model trained with CHH methylation data.

Chromosome	IR64			Azucena		
	*R* ^2^	Correlation	MSE	*R* ^2^	Correlation	MSE
1	0.00	0.63	0.03	0.44	0.67	0.02
2	0.04	0.66	0.03	0.53	0.73	0.01
3	0.37	0.70	0.02	0.49	0.72	0.02
4	0.44	0.72	0.02	0.60	0.81	0.01
5	0.59	0.81	0.02	0.67	0.84	0.01
6	0.44	0.78	0.02	0.68	0.82	0.01
7	0.16	0.53	0.04	0.50	0.73	0.02
8	0.71	0.85	0.01	0.67	0.88	0.02
9	0.32	0.65	0.03	0.50	0.75	0.02
10	0.41	0.70	0.02	0.28	0.69	0.03
11	0.30	0.70	0.02	0.52	0.77	0.01
12	0.54	0.77	0.01	0.35	0.85	0.02

## Data Availability

All the data and Python scripts to apply the model are available at https://github.com/maurope/reCHHombinator (accessed on 3 October 2022).

## Supplementary Materials

The following supporting information can be downloaded at: https://www.mdpi.com/article/10.3390/ijms232012505/s1.
