# Supplementary material for: Methylation in the CHH Context Allows to Predict Recombination in Rice
Source: Int J Mol Sci. 2022 Oct 19;23(20):12505. doi: 10.3390/ijms232012505 (PMC9604423; doi:10.3390/ijms232012505)
Supplement: Supplementary file 1 [file ijms-23-12505-s001.zip › SUPPLEMENTARY MATERIAL_Figures S1_S2_S3.pdf]

International Journal of *Molecular Sciences*

MDPI

SUPPLEMENTARY MATERIAL

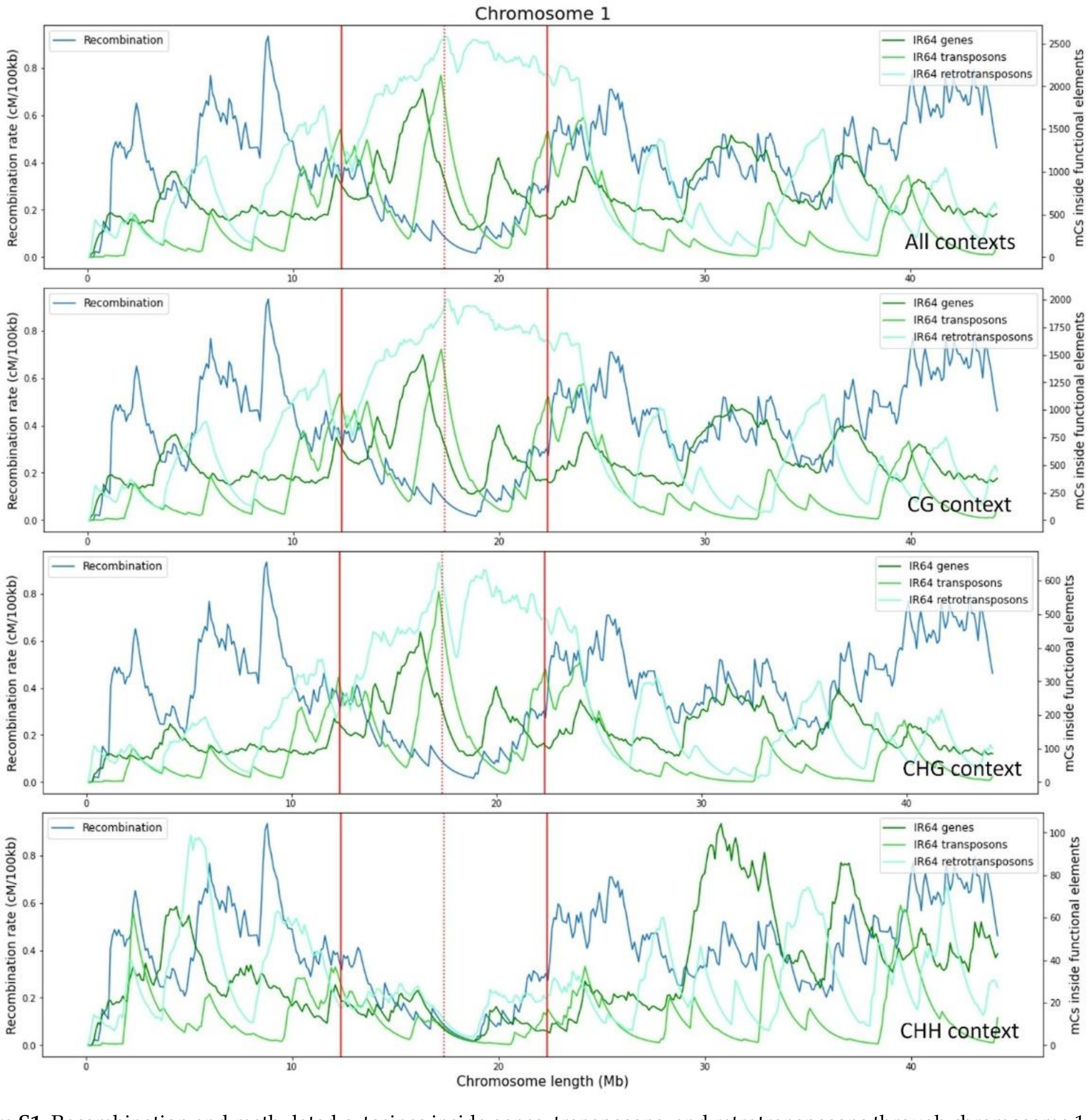

Figure S1. Recombination and methylated cytosines inside genes, transposons, and retrotransposons through chromosome 1 for the rice variety IR64. The centromere is represented by a red dotted line and the influence of the centromere region in recombination by red solid lines.

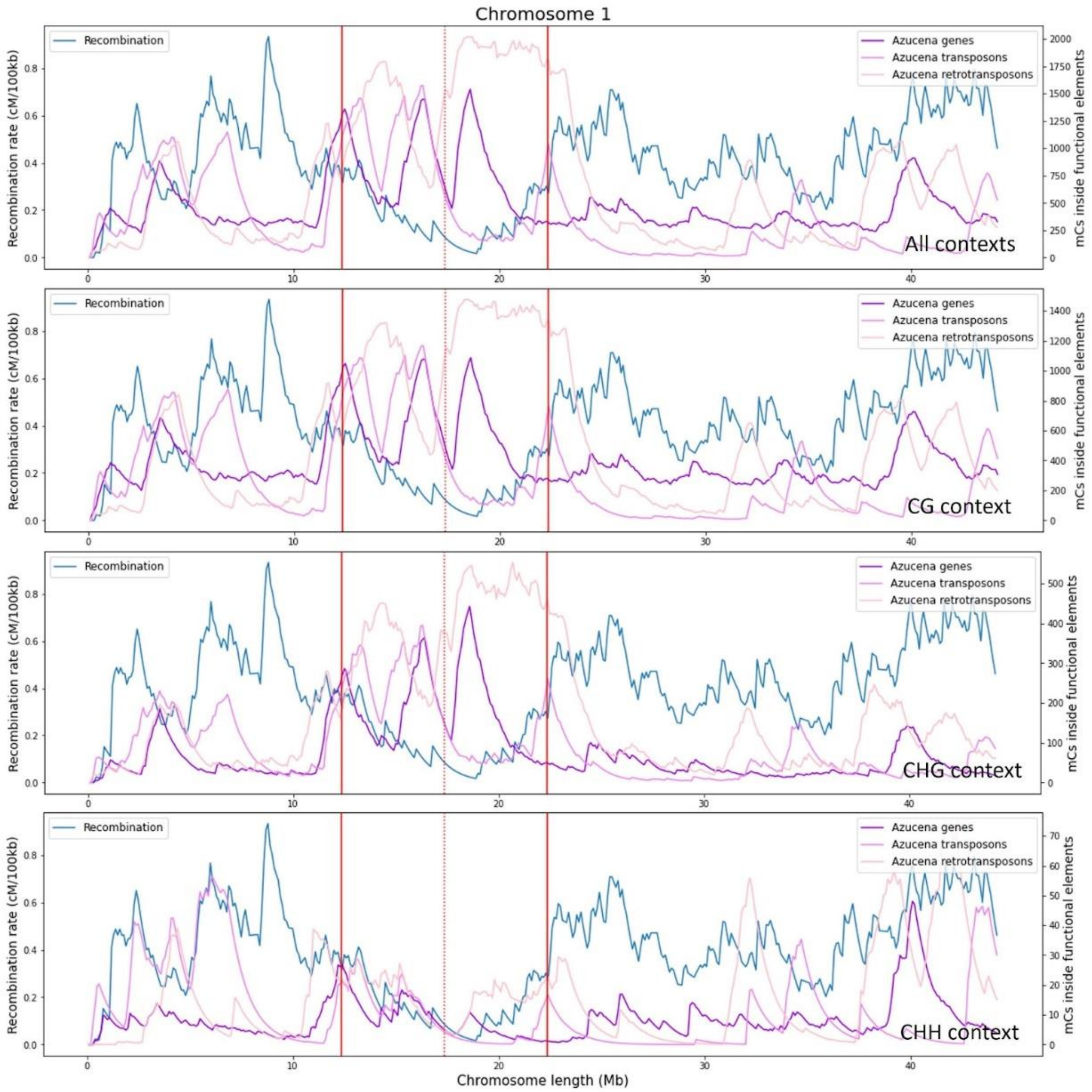

Figure S2. Recombination and methylated cytosines inside genes, transposons, and retrotransposons through chromosome 1 for the rice variety Azucena. The centromere is represented by a red dotted line and the influence of the centromere region in recombination by red solid lines.

All contexts	Complete chromosome						Chromosome arms						Centromere region					
	Genes		Transposons		Retrotransposons		Genes		Transposons		Retrotransposons		Genes		Transposons		Retrotransposons	
Chromosome	IR64	Azucena	IR64	Azucena	IR64	Azucena	IR64	Azucena	IR64	Azucena	IR64	Azucena	IR64	Azucena	IR64	Azucena	IR64	Azucena
1	-0.39	-0.52	-0.24	-0.29	-0.54	-0.14	0.20	0.20	0.01	0.05	0.05	-0.02	-0.03	-0.63	0.28	-0.08	-0.73	0.64
2	-0.43	-0.45	-0.21	0.01	-0.46	-0.54	0.14	0.26	0.23	0.27	0.16	-0.14	-0.73	-0.60	-0.68	0.03	-0.82	-0.36
3	-0.55	-0.61	-0.07	-0.19	-0.62	-0.34	0.04	0.06	0.09	0.16	-0.09	0.04	-0.77	-0.73	0.02	-0.36	-0.78	-0.56
4	-0.71	-0.69	-0.44	-0.56	-0.64	-0.26	-0.39	-0.45	-0.35	-0.44	-0.26	-0.20	-0.68	-0.37	0.01	0.17	-0.83	-0.07
5	-0.69	-0.61	-0.73	-0.24	-0.73	-0.34	0.00	-0.09	-0.09	0.00	-0.18	-0.03	-0.77	-0.56	-0.91	-0.69	-0.79	-0.16
6	-0.57	-0.59	-0.14	0.06	-0.59	0.08	-0.10	-0.06	0.00	0.27	-0.20	0.24	-0.83	-0.81	-0.65	-0.57	-0.83	-0.85
7	-0.41	-0.19	-0.35	-0.28	-0.50	-0.09	0.24	0.48	0.03	0.13	0.00	0.49	-0.44	0.52	-0.04	0.00	-0.16	0.30
8	-0.69	-0.69	-0.65	-0.46	-0.71	-0.36	-0.46	-0.56	-0.52	-0.21	-0.46	-0.11	-0.58	-0.51	-0.33	-0.29	-0.51	-0.78
9	-0.14	-0.27	-0.12	-0.21	-0.29	0.49	-0.31	-0.38	-0.15	-0.22	-0.25	0.44	0.69	0.59	-0.11	0.24	0.63	0.62
10	-0.54	-0.35	-0.46	-0.47	-0.66	-0.56	0.09	-0.01	0.09	-0.08	-0.24	-0.28	-0.73	0.26	-0.33	-0.35	-0.79	-0.64
11	-0.57	-0.35	-0.41	-0.20	-0.50	-0.18	-0.28	-0.12	0.09	0.11	0.00	0.24	-0.70	-0.48	-0.37	-0.17	-0.71	-0.22
12	-0.61	-0.68	-0.61	0.16	-0.65	-0.56	-0.24	-0.47	-0.33	0.40	-0.29	-0.30	-0.52	-0.54	-0.47	0.25	-0.67	-0.81

CG context	Complete chromosome						Chromosome arms						Centromere region					
	Genes		Transposons		Retrotransposons		Genes		Transposons		Retrotransposons		Genes		Transposons		Retrotransposons	
Chromosome	IR64	Azucena	IR64	Azucena	IR64	Azucena	IR64	Azucena	IR64	Azucena	IR64	Azucena	IR64	Azucena	IR64	Azucena	IR64	Azucena
1	-0.36	-0.51	-0.22	-0.35	-0.54	-0.39	0.23	0.19	0.08	0.05	0.07	0.00	0.05	-0.67	0.38	0.12	-0.81	-0.71
2	-0.42	-0.43	-0.20	-0.17	-0.44	-0.57	0.24	0.33	0.26	0.29	0.25	-0.01	-0.79	-0.66	-0.68	-0.23	-0.87	-0.50
3	-0.55	-0.56	-0.09	-0.24	-0.59	-0.52	0.10	0.12	0.18	0.14	0.00	0.00	-0.85	-0.70	0.11	-0.29	-0.80	-0.72
4	-0.68	-0.68	-0.36	-0.67	-0.63	-0.58	-0.35	-0.43	-0.23	-0.53	-0.27	-0.40	-0.59	-0.53	0.28	0.05	-0.81	-0.24
5	-0.67	-0.63	-0.74	-0.55	-0.71	-0.54	0.00	-0.07	-0.11	-0.14	-0.14	-0.09	-0.78	-0.57	-0.90	-0.52	-0.82	-0.29
6	-0.54	-0.56	-0.11	-0.16	-0.54	-0.19	0.01	0.10	0.07	0.21	-0.04	0.27	-0.86	-0.80	-0.70	-0.69	-0.87	-0.87
7	-0.39	-0.23	-0.32	-0.29	-0.45	-0.26	0.24	0.46	0.09	0.15	0.12	0.38	-0.46	0.33	-0.20	0.19	-0.20	0.23
8	-0.66	-0.71	-0.58	-0.45	-0.72	-0.55	-0.39	-0.52	-0.45	-0.22	-0.47	-0.22	-0.59	-0.55	-0.14	-0.13	-0.57	-0.80
9	-0.21	-0.40	-0.13	-0.32	-0.34	0.05	-0.35	-0.38	-0.18	-0.22	-0.28	0.13	0.70	0.52	-0.09	0.13	0.61	0.59
10	-0.49	-0.38	-0.47	-0.58	-0.63	-0.61	0.11	0.02	-0.01	-0.19	-0.26	-0.27	-0.72	0.18	-0.14	-0.61	-0.80	-0.73
11	-0.55	-0.24	-0.43	-0.23	-0.47	-0.27	-0.23	-0.04	-0.04	0.07	0.00	0.18	-0.82	-0.22	-0.15	-0.06	-0.84	-0.25
12	-0.58	-0.65	-0.54	0.18	-0.61	-0.56	-0.19	-0.40	-0.31	0.45	-0.25	-0.27	-0.61	-0.61	-0.24	0.41	-0.58	-0.80

CHG context	Complete chromosome						Chromosome arms						Centromere region					
	Genes		Transposons		Retrotransposons		Genes		Transposons		Retrotransposons		Genes		Transposons		Retrotransposons	
Chromosome	IR64	Azucena	IR64	Azucena	IR64	Azucena	IR64	Azucena	IR64	Azucena	IR64	Azucena	IR64	Azucena	IR64	Azucena	IR64	Azucena
1	-0.47	-0.58	-0.27	-0.18	-0.56	-0.20	0.18	0.02	0.03	0.04	0.00	-0.05	-0.49	-0.62	0.17	-0.08	-0.75	-0.63
2	-0.52	-0.55	-0.25	-0.21	-0.52	-0.58	0.05	0.06	0.15	0.19	0.05	-0.24	-0.77	-0.57	-0.78	-0.35	-0.87	-0.22
3	-0.62	-0.62	-0.22	-0.11	-0.64	-0.28	-0.07	-0.09	-0.11	0.08	-0.08	0.10	-0.79	-0.62	-0.02	0.06	-0.79	-0.51
4	-0.73	-0.72	-0.44	-0.42	-0.69	-0.12	-0.40	-0.51	-0.42	-0.24	-0.32	-0.08	-0.75	-0.42	-0.04	0.34	-0.83	-0.10
5	-0.74	-0.65	-0.74	-0.23	-0.78	-0.27	-0.14	-0.20	-0.15	0.03	-0.32	-0.05	-0.80	-0.63	-0.88	-0.29	-0.78	-0.22
6	-0.61	-0.63	-0.26	0.00	-0.65	0.17	-0.17	-0.35	-0.04	0.31	-0.30	0.28	-0.82	-0.75	-0.75	-0.74	-0.84	-0.85
7	-0.46	-0.26	-0.35	-0.30	-0.51	-0.09	0.17	0.24	0.07	0.04	0.05	0.47	-0.34	0.61	-0.24	0.20	-0.17	0.16
8	-0.67	-0.69	-0.69	-0.51	-0.68	-0.37	-0.44	-0.65	-0.58	-0.28	-0.39	-0.11	-0.42	-0.54	-0.43	-0.11	-0.58	-0.79
9	-0.13	-0.30	-0.25	-0.19	-0.38	0.68	-0.27	-0.35	-0.18	-0.21	-0.24	0.60	0.74	0.57	-0.27	0.34	0.63	0.61
10	-0.62	-0.46	-0.52	-0.48	-0.68	-0.55	0.06	-0.10	0.06	-0.10	-0.24	-0.23	-0.80	0.05	-0.54	-0.60	-0.78	-0.70
11	-0.64	-0.42	-0.47	-0.17	-0.46	0.00	-0.36	-0.27	0.06	0.22	0.16	0.41	-0.80	-0.52	-0.54	-0.43	-0.70	0.12
12	-0.66	-0.73	-0.46	0.18	-0.66	-0.45	-0.41	-0.56	-0.06	0.49	-0.35	-0.25	-0.53	-0.51	-0.13	0.34	-0.59	-0.78

CHH context	Complete chromosome						Chromosome arms						Centromere region					
	Genes		Transposons		Retrotransposons		Genes		Transposons		Retrotransposons		Genes		Transposons		Retrotransposons	
Chromosome	IR64	Azucena	IR64	Azucena	IR64	Azucena	IR64	Azucena	IR64	Azucena	IR64	Azucena	IR64	Azucena	IR64	Azucena	IR64	Azucena
1	0.62	0.19	0.20	0.16	0.47	0.27	0.29	0.10	-0.03	0.03	0.14	0.24	-0.49	-0.62	0.17	-0.08	-0.75	-0.63
2	0.61	0.39	0.56	0.11	0.59	0.49	0.31	0.44	0.44	-0.11	0.38	0.40	-0.77	-0.57	-0.78	-0.35	-0.87	-0.22
3	0.46	0.34	0.35	0.00	0.46	0.08	0.18	0.26	0.33	-0.18	0.28	0.06	-0.79	-0.62	-0.02	0.06	-0.79	-0.51
4	0.48	0.53	0.11	0.00	0.70	0.38	0.30	0.51	-0.02	0.01	0.57	0.46	-0.75	-0.42	-0.04	0.34	-0.83	0.10
5	0.84	0.48	0.48	0.13	0.79	0.52	0.66	0.24	0.22	-0.02	0.57	0.33	-0.80	-0.63	-0.88	-0.29	-0.78	-0.22
6	0.71	0.58	0.55	-0.02	0.75	0.03	0.54	0.40	0.36	0.08	0.63	-0.09	-0.82	-0.75	-0.75	-0.74	-0.84	-0.85
7	0.36	0.48	-0.09	0.29	0.29	0.38	-0.05	0.24	-0.36	0.34	-0.05	0.25	-0.34	0.61	-0.24	0.20	-0.17	0.16
8	0.85	0.30	0.59	0.15	0.70	0.63	0.79	0.07	0.46	0.22	0.56	0.53	-0.42	-0.54	-0.43	-0.11	-0.58	-0.79
9	0.70	0.61	0.72	0.27	0.62	0.45	0.48	0.48	0.57	0.14	0.40	0.40	0.74	0.57	-0.27	0.34	0.63	0.61
10	0.63	0.71	0.24	0.13	0.51	0.28	0.47	0.69	0.32	0.23	0.25	-0.05	-0.80	0.05	-0.54	-0.60	-0.78	-0.70
11	0.62	0.48	0.20	0.26	0.43	0.04	0.26	0.15	0.03	-0.01	-0.02	-0.14	-0.80	-0.52	-0.54	-0.43	-0.70	0.12
12	0.69	0.21	0.28	0.14	0.65	0.80	0.61	0.09	0.03	0.03	0.46	0.76	-0.53	-0.51	-0.13	0.34	-0.59	-0.78

Figure S3. Correlations between recombination rates and the count of methylated cytosines in genes, transposons and retrotransposons rates for rice varieties IR64 and Azucena for each methylation context. Blue and red colors correspond to positive and negative correlations, respectively. The higher the correlation value, the higher the color intensity.
